# Antibiotic practices among household members and their domestic animals within rural communities in Cumilla district, Bangladesh: a cross-sectional survey

**DOI:** 10.1186/s12889-021-10457-w

**Published:** 2021-02-25

**Authors:** Joseph Paul Hicks, Sophia M. Latham, Rumana Huque, Mahua Das, Jane Newell, S. M. Abdullah, Zunayed Al Azdi, Ishrat Jahan, Christian Rassi, Prudence Hamade, Muhammad Shafique, Mohammad Saiful Islam, Rebecca King

**Affiliations:** 1 Nuffield Centre for International Health and Development, University of Leeds, Leeds, LS2 9JT UK; 2 Department of Livestock and One Health, Institute of Infection, Veterinary and Ecological Sciences, University of Liverpool, Leahurst Campus, Neston, Cheshire, CH64 7TE UK; 3 ARK Foundation, Suite C-3 & C-4, House # 06, Road # 109, Gulshan-2, Dhaka, 1212 Bangladesh; 4 Department of Economics, University of Dhaka, Dhaka, 1000 Bangladesh; 5 University of Liverpool Medical School, Cedar House, Ashton St, Liverpool, L69 3GE UK; 6 Malaria Consortium, The Green House, 244-254 Cambridge Heath Road, London, E2 9DA UK; 7 Faculty of Surgery and Professor of Paediatric Surgery, Bangabandhu Sheikh Mujib Medical University, Dhaka, Bangladesh

## Abstract

**Background:**

Antibiotic resistance is a global threat to human health, and inappropriate use of antibiotics in humans and animals is widely considered to be a key driver of antibiotic resistant infections. Antibiotic use in humans and animals is growing rapidly in low- and, particularly, middle-income countries. However, there is little detailed understanding about practices related to the use of antibiotics in humans and animals within community settings in such countries. Here we aimed to understand the antibiotic practices of rural households across Cumilla district, Bangladesh, in relation to household members and their domestic animals.

**Methods:**

In 2018 we conducted a cross-sectional survey using representative cluster sampling methods. We collected self-reported information from 682 female and 620 male household heads, with women also asked about their children’s antibiotic practices.

**Results:**

Only 48% (95% CI: 40, 56%) of women and men had heard of antibiotics, and among those women and men who were aware of antibiotics and the children of those women 70% (95% CI: 64, 76%) reported having previously taken antibiotics, while among these individuals who reported previously taking antibiotics 21% (95% CI: 18, 25%) said they had done so most recently within the last month. Risky/inappropriate antibiotic practices in humans and animals were often reported. For example, among women and men who were aware of antibiotics and the children of those women 52% (95% CI: 40, 63%) reported previously taking antibiotics for a “cough/cold”, despite antibiotics being typically inappropriate for use against viral upper respiratory tract infections. Among poultry-owning respondents who were aware of antibiotics 11% (95% CI: 8, 15%) reported previously giving healthy poultry antibiotics, mainly for growth/prophylaxis, while among cattle-owning respondents who were aware of antibiotics and reported previously giving their cattle feed 20% (95% CI: 9, 37%) said the feed had contained antibiotics at least sometimes.

**Conclusions:**

Our results highlight the need for context-adapted interventions at both the community level and the health systems level to reduce inappropriate antibiotic use among humans and domestic animals in rural Bangladesh. Successfully reducing inappropriate use of antibiotics among humans and animals is a required and critical step in tackling antimicrobial resistance.

**Supplementary Information:**

The online version contains supplementary material available at 10.1186/s12889-021-10457-w.

## Introduction

Antibiotic resistance (ABR) is recognised as one of the biggest global challenges for future human health and development, as shown by the creation of global action plans by international institutions such as the World Health Organization (WHO), World Organisation for Animal Health (OIE), and the Food and Agriculture Organization of the United Nations (FAO) [1]. Globally, antibiotic resistant microbes are being recorded at increasing rates [2], with ABR believed to be caused largely by the inappropriate use of antibiotics in both humans and animals [3]. Although antibiotic consumption in humans and animals is still typically highest in high-income countries, in recent decades there have been substantial increases within low- and middle-income countries (LMICs) [2, 4–6]. Within LMICs most inappropriate use of antibiotics by humans probably results from the inappropriate prescribing and sale of antibiotics, mainly via poorly regulated private providers, for conditions that do not require antibiotics, particularly uncomplicated upper respiratory tract infections (URTIs) [7, 8]. Most inappropriate use of antibiotics in animals within LMICs comes from their indiscriminate use as growth promoters in large-scale commercial farming [2, 6, 9]. However, in LMICs very small-scale, household-based, domestic or “backyard” animal farming is an extensive and important source of food and income across rural communities [10]. For example, domestic/household poultry farming accounts for the majority of the poultry populations in many LMICs [11].

Many academic studies and global action initiatives have therefore called for a better understanding of how antibiotics are being used in humans and animals in LMICs, particularly in rural settings where most LMIC populations live [9]. Looking at both human and animal antibiotic practices is also a key part of a holistic “One Health” approach to tackling ABR, as recommended by the WHO, OIE and FAO [1]. Currently though, most survey work in this area has only addressed human or animal use separately, most human-focused studies have been facility- or provider-based, and most animal-focused studies have only addressed commercial, large-scale farming (e.g. [8, 12, 13]). However, only community-based studies can provide a representative understanding of antibiotic practices in humans at the community-level due to the inherent selection bias of facility- or provider-based studies [14]. Community-based studies also offer the most obvious approach to explore antibiotic practices in small-scale, domestic animal farming, which has received very little attention.

Therefore, in this study we surveyed rural households in villages across a typical district within Bangladesh, a lower-middle-income country [http://data.worldbank.org/about/country-and-lending-groups]. Our main aim was to understand rural household practices in relation to antibiotic use in humans and households’ domestic animals, to understand the prevalence of key potential drivers of ABR in this context.

## Methods

### Setting and participants

We conducted our cross-sectional, rural household survey across Cumilla district, Bangladesh, between May and June 2018, but we excluded the two predominantly urban sub-districts of Sadar and Sadar Dakshin leaving 14 mainly rural sub-districts. Cumilla had an estimated population of 5.4 million in 2011, and has an economy that is based primarily on agricultural production and agricultural product processing, mainly via cottage industries [15]. As is typical across rural Bangladesh, subsistence farming, including small-scale, household-based animal husbandry, is common [16]. This typically involves keeping small numbers of poultry, mainly chickens and ducks, and/or cattle, goats and sheep. Within selected households we interviewed, subject to consent, both the self-identified female and male household head if aged between 18 and 49.

### Questionnaire and survey topics

We collected all data using a structured paper questionnaire with Bengali-language based questions. The questions were informed by 1) related surveys [17–19], 2) WHO, FAO and OIE recommended best practices [20], and 3) our previous qualitative exploratory work and small-scale quantitative survey work in this context, which we did as part of a study that informed the development of a community educational intervention aimed at improving the appropriate use of antibiotics in humans and animals. They were then refined via two-rounds of iterative formative field work involving qualitative exploratory work and pilot testing of the survey methods and questionnaire. Our questionnaire collected basic socio-demographic data questions, and we then asked a screening question to check whether respondents “had heard of antibiotics?” We knew from our formative field work and the contextual knowledge of our Bangladeshi colleagues that there is no widely used Bengali term for “antibiotic”, and that those who are aware of antibiotics typically use the English word “antibiotic”, and we therefore used this term. To understand antibiotic practices within households we then asked female and male respondents about their own antibiotic practices, including if and when they had previously used antibiotics, where they had obtained them from, what illnesses had caused them to obtain them, and a series of questions related to the WHO’s, FAO’s and OIE’s recommended best practices when accessing and using antibiotics [20]. If female respondents had any children under 15 we also asked them these same questions but in relation to their own children. We also asked female and male respondents about animal ownership, animal husbandry practices, animal health and treatment, and animal-related antibiotic practices within their household. Our formative field work and contextual knowledge indicated that poultry are typically looked after by women, while cattle, goats or sheep are typically looked after by men. Therefore, we only asked women these questions in relation to any household poultry, and we only asked men these questions in relation to any household cattle, goats or sheep.

### Sampling and interviews

We used a two-stage cluster sampling approach and stratified our sampling by sub-district [21]. In the first stage of sampling we used data from the most recent (2011) Bangladeshi census to randomly select, with probability proportional to size, two villages per sub-district. In the second stage of sampling seven field teams, each consisting of two interviewers, visited each selected village in turn, on a sub-district by sub-district basis, and used a compact segmented sampling approach to select households. This involved drawing a rough sketch map of the village, including all major boundaries and the location of approximately all households, and then dividing the map into segments each containing approximately 25 households. Once drawn, the interviewers then sent digital photos of the maps back to the research team in Dhaka city via the internet, and the research team then numbered each segment from 1 to n, starting from the most northerly segment and working in a clockwise direction, before using a random number generator to select one of the mapped segments. The research team then told the field teams which segment had been selected, and then the field teams approached every potential household building in the selected segment and attempted to interview both the self-identified female and male household head of each household separately. If a female and/or a male household head was not immediately available the field team arranged a re-visit where possible. This was attempted up to three times before the respondent was considered a non-responder. We did not replace non-responders or non-consenters. This sampling process results in an equal probability of selection for households/respondents [21], meaning that unbiased results can be estimated without the use of weights. However, due to the multi-stage clustered sampling process the non-independence of households/respondents within clusters must be accounted for during analysis to ensure accurate coverage of confidence intervals.

### Sample size

We calculated our sample size based on a generic binary outcome because almost every question in our questionnaire would result in categorical responses where each category level (e.g. yes/no/don’t know) can be treated as a separate binary outcome, with the proportion/percentage of respondents providing each category-level response estimated along with its 95% confidence interval. Based on logistical resources considerations and desired levels of precision, we estimated that if we sampled 720 households, and therefore 720 women and 720 men (assuming one female and one male household head per household), then we would have a sufficient sample size to estimate any binary outcome, separately for women or men, with a precision (95% confidence interval width) of 6.9 percentage points or less [22]. This estimate assumed a design effect of 1.8, which was rounded up from the mean design effect across all key indicators in the 2014 Bangladesh DHS report, and an overall response rate of 95%, which was based on the mean of the response rates for the female and male Bangladesh DHS surveys [23]. It also assumed that the mean/proportion of the generic binary variable was 0.5, as this results in the largest possible sample size for a binary outcome and is therefore the most conservative assumption. Given that we planned to sample individuals from two villages in 14 sub-districts this meant we needed to sample 25 women and 25 men per village, or 1440 women and men in total.

### Data collection

During field work the research team monitored progress remotely, and the field teams returned to the research team offices every week to hand over the questionnaire data sheets, which the research team then checked 5% of for data quality. After data collection the research team trained separate data entry staff, who entered all data from the paper questionnaires into a specially designed SPSS database, which was then checked for obvious errors and anomalies and cleaned by both the Bangladeshi research team and then checked again by JPH.

### Statistical analyses

We produced descriptive statistics to describe the key sociodemographic characteristics of the sample, and we produced inferential statistics in the form of point estimates and 95% confidence intervals to draw conclusions about responses given to questions. These were either in the form of percentages and their associated 95% confidence intervals for categorical questions, i.e. questions where the responses were categorical, or in the form of means and their associated 95% confidence intervals in the very few cases of numerical questions, i.e. questions where the responses were numerical. Depending on the question the estimated response percentages were specific to either female respondents, male respondents, the children of female respondents, household poultry, household cattle, or household goats and/or sheep (collectively).

For all categorical questions the 95% confidence intervals were calculated using a “logit” method that involved fitting a logistic regression model before computing a Wald-type interval on the log-odds scale, which was then transformed to the probability-scale. For categorical questions related to human antibiotic practices we also produced “overall” estimates based on combining the responses from all female and male respondents, and, if applicable, the responses of female respondents in relation to their children. We also estimated how responses to categorical questions differed between women, men and children, or between the different animal groups, as appropriate to the question. We did this by treating each response category (e.g. “where did you last obtain antibiotics?”: “from a pharmacy”) as an individual binary outcome (i.e. response selected/not selected by responder) and using a binomial regression model [24] we estimated the unadjusted absolute difference in the percentage of respondents selecting that response between each relevant group (e.g. women compared to men), i.e. the unadjusted percentage point difference.

We used R [25] statistical software (version 3.5.2) for all analyses and accounted for the clustered and stratified sampling features of the survey using Taylor series linearisation methods for analysing complex surveys, implemented in the R *survey* [26, 27] and *sryvr* [28] packages, with subpopulation analyses appropriately including the original survey design information [29]. Where subpopulation analyses resulted in “lonely sampling clusters”, where only a single cluster remains within a stratum, we used the *survey* package’s “average” option, where “the stratum contribution to the variance is taken to be the average of all the strata with more than one PSU.” [26, 27] In all analyses we excluded any cases that had missing outcomes.

## Results

Between May and June 2018, across the 14 chosen subdistricts of Cumilla we selected 28 villages (two per subdistrict) and household clusters (one per village). Across the selected households there were 691 eligible female household heads and 672 eligible male household heads, with 682 (98.7%) and 620 (92.3%) female and male household heads agreeing to participate respectively. Female and male respondents’ ages were broadly evenly distributed across the eligible age range, both were mostly married (97%), and a substantial proportion (37%) of both either had no education or had not completed primary education. However, while 74% of men reported having worked for cash or in-kind payment in the last month only 20% of women did so (Table 1).

**Table 1:** Respondent characteristics (*n* = 1302)

Respondent characteristics	Overall	Men	Women
Sex			
Female	52% (682)	–	–
Male	48% (620)	–	–
Age			
18–27	33% (423)	21% (128)	44% (295)
28–37	33% (422)	33% (203)	32% (219)
38–49	34% (439)	45% (276)	24% (163)
Education status			
No education/incomplete primary	37% (484)	40% (245)	35% (239)
Completed primary/incomplete secondary	43% (564)	38% (237)	48% (327)
Completed secondary	15% (195)	16% (99)	14% (96)
More than secondary	4% (54)	6% (35)	3% (19)
Marriage status			
Married	97% (1249)	97% (586)	97% (663)
Never married	1% (15)	2% (15)	0% (0)
Widowed/divorced/separated	2% (24)	1% (6)	3% (18)
Worked for cash/in-kind payment in last month?			
Yes	45% (589)	74% (453)	20% (136)
No	55% (707)	26% (162)	80% (545)

Values are % (n)

Below we highlight the responses to key questions. For questions related to human antibiotic practices we only highlight the overall results unless there were clear differences between groups. For simplicity we sometimes refer to children as “reporting” their responses, but technically their mothers reported on their behalf.

### Household practices related to antibiotic purchase and use in household members

Overall 48% (95% CI: 40, 56%) of women and men said they had heard of antibiotics, and we refer to these individuals as being “antibiotic-aware” from here on. Among these antibiotic-aware women and men 80% (95% CI: 76, 84%) and 76% (95% CI: 68, 82%), respectively, reported having ever taken antibiotics, while 56% (95% CI: 45, 67%) of antibiotic-aware women with children under 15 reported that at least one of their children had ever taken antibiotics. Across all women, men and children who reported taking antibiotics overall 21% (18, 25%) reported having taken antibiotics within the last month. See Table S1 for full results on reported antibiotic awareness and household member antibiotic use.

We asked those antibiotic-aware respondents who reported that they or any of their children had previously taken antibiotics what illnesses or main symptoms they had ever taken antibiotics for. Overall the most frequently reported condition was undifferentiated “fever” (68% [95% CI: 60, 75%]) followed by “cough/cold” (52% [95% CI: 40, 63%]). For all other illnesses/main symptoms the overall results were substantially less than 50%, but notably “sore throat”, a common symptom of viral URTIs, was reported by 24% (95% CI: 19, 30%). See Table S2 for full results on reported reasons for obtaining antibiotics for human use. We also asked this same group of respondents where they had ever obtained antibiotics from. Overall by far the most frequently reported source was a pharmacy (79% [95% CI: 73, 84%]), followed by a private medical practitioner (35% [95% CI: 27, 44%]) and a paramedic/village doctor (20% (95% CI: 14, 27%]). Overall only 5% (95% CI: 2, 12%) reported ever obtaining antibiotics from the only local source of free primary care and antibiotics: a Community Clinic. See Table S3 for full results on reported sources for obtaining antibiotics for human use. This same group of respondents did not frequently report practices when last taking antibiotics that were contrary to the WHO’s recommended best practices [20], which are: 1) Only use antibiotics when prescribed by a certified health professional. 2) Never demand antibiotics if your health worker says you don’t need them. 3) Always follow your health worker’s advice when using antibiotics. 4) Never share or use leftover antibiotics. Overall the most frequently reported inappropriate behaviours were obtaining an antibiotic without a prescription (30% [95% CI: 24, 37%]) followed by “taking fewer antibiotics than recommended by a health professional” (17% [95% CI: 12, 23%]). See Table S4 for full results on antibiotic-use related practices that are consistent/inconsistent with the WHO’s recommended best practices.

### Household practices related to antibiotic resistance and antibiotic use in domestic animals

73% (95% CI: 68, 78%) of households reported owning poultry (mean poultry if any owned = 10 [95% CI: 9, 11]), mainly chickens and ducks, while 32% (95% CI: 26, 40%) reported owning cattle (mean cattle if any owned = 2 [95% CI: 2, 3]), and 9% (95% CI: 6, 13%) reported owning goats and/or sheep (mean goats/sheep if any owned = 3 [95% CI: 2, 4%]). See Table S5 and S6 for full ownership results. Due to the low numbers owning goats and/or sheep we only present results for poultry and cattle. Animal husbandry practices that may drive zoonotic disease transmission, thereby increasing the need for antibiotics, were frequently reported. Specifically, 76% (95% CI: 68, 83%) and 35% (95% CI: 28, 43%) of poultry- and cattle-owning households, respectively, reported that they kept their poultry/cattle in their houses at night, while 42% (95% CI: 35, 49%) and 49% (95% CI: 35, 64%) of poultry- and cattle-owning households, respectively, reported that their poultry/cattle sometimes drank from or bathed in the household’s drinking or cooking water source(s). See Table S7 for full results related to animal husbandry practices relevant to antibiotic resistance and antibiotic use in domestic animals.

Animal illness was frequently reported, particularly for poultry. Among poultry-owning households 65% (95% CI: 53, 75%) reported at least one of their poultry had previously been ill or died suddenly, and among poultry-owning households reporting previous poultry illness 77% (95% CI: 69, 84%) reported that the most recent illness was within the last 6 months. Among cattle-owning households 27% (95% CI: 17, 39%) reported at least one of their cattle had previously been ill or died suddenly, and among cattle-owning households reporting previous cattle illness 57% (95% CI: 39, 73%) reported that the most recent illness was within the last 6 months.

Ill cattle appear more likely to be treated than ill poultry. Among animal-owning households that reported ever previously seeking treatment for ill animal(s) 75% (95% CI: 60, 86%) of these households that owned poultry and 98% (95% CI: 79, 100%) of these households that owned cattle reported that the treatment involved either “western medicines” (in the form of either pills, liquids or injections) or “food with added medicine/drugs”. Among poultry-owning households that reported ever treating their animal(s) when they were ill only 1% (95% CI: 0, 7%) and 4% (95% CI: 1, 18%) respectively, reported having previously used a government or private veterinarian, while the most frequently reported previous source of treatment was a drug seller/pharmacist (48% [95% CI: 35, 60%]). However, among cattle-owning households that reported ever treating their animal(s) when they were ill 25% (95% CI: 14, 41%) and 32% (95% CI: 12, 62%), respectively, reported having previously used a government or private vet, although 14% (95% CI: 5, 31%) still reported previously using a drug seller/pharmacist, and 27% (95% CI: 19, 38%) a village doctor/paramedic/private medical practitioner. See Table S8 for full results related to domestic animal illness and treatment.

In relation to antibiotics, we asked antibiotic-aware respondents of animal-owning households who reported previously having ill animal(s) treated whether such treatment ever involved antibiotics, and this was reported by 24% (95% CI: 11, 45%) of these households that owned poultry and 36% (95% CI: 13, 67%) of these households that owned cattle. We also asked antibiotic-aware respondents if any of their animals had ever been given antibiotics when they were not ill, and this was reported by 11% (95% CI: 8, 15%) of poultry-owning households and 2% (95% CI: 0, 9%) of cattle-owning households. Additionally, 14% (95% CI: 9, 23%) of antibiotic-aware poultry-owning households said they “sometimes/usually/always” bought commercial feed for their animals, and 21% (95% CI: 7, 50%) of those who responded in this way said that this feed “sometimes/usually/always” contained antibiotics. For antibiotic-aware cattle-owning households the corresponding figures were 63% (95% CI: 41, 81%) and 20% (95% CI: 9, 37%) respectively (Table S5). Unfortunately, the sample sizes for responses to subsequent questions related to animal antibiotic use, including when healthy and in feed, were too small to be of much inferential use, but reported sources of antibiotics included drug sellers/pharmacists and village doctors/paramedics/private medical practitioners, and reasons for giving healthy animals antibiotics included increasing growth or production of eggs/milk/meat and preventing disease. See Table S8 for full results on practices related to antibiotic-use in domestic animals. We also asked respondents about domestic animal vaccination and related issues and present the results for interested readers in Table S9.

## Discussion

To our knowledge, our study provides the first comprehensive information from Bangladesh about rural households’ antibiotic practices in relation to humans and domestic animals. We found a substantial lack of awareness about the existence of antibiotics as a distinct class/type of medicine in this context, which necessarily restricted our ability to understand antibiotic practices among non-aware individuals. This problem is faced by all similar surveys of rural LMIC communities, with levels of awareness varying substantially but typically being moderate to low [30–32], although comparisons are difficult. Some similar surveys do not initially check awareness and apparently rely on respondents who are not aware of antibiotics to select “don’t know” type responses [19, 33, 34], which may lead to increased bias. Therefore, our results, and those of similar studies looking at antibiotic knowledge in more detail, highlight that there is a huge amount of work to be done on educating rural populations in LMICs about antibiotics and appropriate practices.

Like similar surveys from other LMICs, including those in urban settings, our data indicate high levels of antibiotic consumption among rural community members [17, 33–36]. Globally, national-level rates of antibiotic resistant microbes/infections are closely correlated with human antibiotic consumption rates [37, 38]. Therefore, reducing inappropriate antibiotic use in Bangladesh and other LMICs is required to tackle ABR as overall antibiotic use is expected to continue to rise across LMICs. Probably the most frequent cause of inappropriate antibiotic use for human illness is when antibiotics are taken for URTIs. Globally, URTIs are likely responsible for most visits to formal and informal healthcare providers, but because URTIs are usually viral and self-limiting international guidance advises against routinely treating them with antibiotics unless there are complications or other clear reasons [39, 40]. However, our results suggest that antibiotic consumption for URTIs is probably common among adults and children in these communities. This is consistent with data from other LMIC community settings as well as formal primary care and informal/private healthcare provider settings in both LMICs and high income countries (HICs) [13, 41–48]. Evidence from both LMICs and HICs shows how important patients’ and caregivers’ beliefs, expectations and demands are in determining whether healthcare providers prescribe/sell antibiotics inappropriately, which highlights that the public have a vital role to play in tackling inappropriate antibiotic use among humans [48–52].

However, despite this clear evidence from across the world that highlights just how problematic inappropriate antibiotic prescribing is within formal and informal primary care settings, appropriately used antibiotics are obviously critical and irreplaceable tools that can substantially reduce mortality from communicable diseases within LMICs, particularly among children. And yet in most LMICs there is still actually often a lack of access to appropriate antibiotics in primary care settings [8]. There is also limited but compelling evidence that mass, untargeted distribution of some antibiotics can reduce mortality in LMICs [53, 54]. Therefore, LMICs must somehow find a balance between increasing appropriate access to antibiotics for human use while reducing inappropriate use [55], but this will be difficult to achieve without affordable and effective diagnostic tools for diseases such as URTIs, appropriate training of healthcare providers, and effective public education. Encouragingly though, in some LMIC primary care settings multi-component interventions that target providers and patients/caregivers, via approaches such as provider training, peer-review of antibiotic prescribing rates, and provider-led patient/caregiver education, have led to substantial and sustained reductions in inappropriate prescribing [56, 57].

We found that the main reported sources for antibiotics were private providers, particularly pharmacies, which in practice may also mean informal and unregulated drug sellers, again consistent with similar surveys [14, 32, 35]. It is well established that most medicines in LMICs are obtained from private sources, which are typically far more numerous, easily accessible and often preferred to public facilities, despite often lacking regulation and providing low-quality care, including the frequent sale of antibiotics for inappropriate or incorrectly diagnosed conditions [58]. We were surprised that very few individuals reported previously obtaining antibiotics from Community Clinics though, given there is approximately one Community Clinic for every 6000 individuals in rural areas of Bangladesh, and given they provide free, basic primary-level care, including providing some antibiotics for non-severe conditions. Despite previous challenges with the quality of care being provided in Community Clinics, a training programme for Community Clinic nurses demonstrated high levels of appropriate antibiotic provision and has now been scaled-up nationally [59]. Therefore, if Bangladesh can find a way to encourage individuals to use Community Clinics more frequently this could be a cost-effective way to reduce inappropriate antibiotic use within rural communities.

Antibiotic-aware respondents who reported using antibiotics did not report deviating from the WHO’s recommended best practices very frequently. For example, most such respondents reported that they and/or their children purchased antibiotics most recently using a prescription. However, contextual knowledge along with studies from Bangladesh and similar contexts suggest that prescriptions are rarely required to obtain medicines from private sources [60–62]. Similar surveys have asked comparable questions but typically find that inappropriate practices are reported more frequently than here [17, 19, 63]. However, it is not clear whether this reflects differences in practices or just the effects of reporting bias, and further research is required to explore this question.

Animal-owning respondents frequently reported previous experience of animal illness, and that treatments primarily involved “western medicines” or “drugs”. They also reported seeking treatment for ill cattle far more often than ill poultry, probably reflecting the far higher value of cattle. Evidence from rural areas within another Bangladeshi district suggests such generic terms might sometimes/often reflect the use of antibiotics, but this was based on small numbers of qualitative interviews [64]. As there is very little other relevant data, the extent of therapeutic antibiotic use in domestic animals within Bangladesh or other LMICs remains unclear. We also found that human healthcare providers were reportedly frequently used to treat ill poultry, and although veterinary care was reportedly used much more frequently for ill cattle human-healthcare providers were still commonly used. This is also consistent with Roess et al. [64] and studies from other LMICs indicating that human healthcare providers are often used for sick animals [65, 66], and reinforces the view that LMICs face a huge challenge in providing and facilitating access to appropriate and qualified healthcare for domestic animals.

We also found some evidence of limited use of antibiotics in healthy poultry or cattle, potentially to increase growth or production of eggs/milk/meat or as prophylactics. There is clear and growing evidence linking the indiscriminate use of antibiotics within small- and large-scale agriculture to the development of antibiotic resistant microbes [67–69], and there is an increasing trend in the use of antibiotics for growth promotion and prophylaxis in commercial agriculture across LMICs. However, there has been very little focus on domestic animal husbandry, as opposed to small-scale farming [69], despite its prevalence in LMICs. For example, in Bangladesh 55% of households own chickens and 33% own cattle, but 76% of chicken owners have ≤10 birds and 83% of cattle owners have ≤3 cattle [16]. Therefore, while the use of antibiotics in domestic animals for non-therapeutic reasons may not yet be common in this context, given the scale of domestic animal ownership in Bangladesh any increases in such practices would represent clear risks for rural Bangladeshi communities given the inevitable increase in selection pressure for antibiotic resistant microbes/infections [67–69]. Consistent with related studies [64], we also found risky animal husbandry practices were frequently reported by animal-owning households, like sharing drinking/bathing water sources with animals. Such practices are known to increase the transmission of microbes among household members, and therefore represent pathways by which – potentially increasingly prevalent – antibiotic resistant microbes could infect rural communities.

A key limitation of our study is that we had to restrict our inferences for most questions to the sub-populations who were able to answer them, particularly the sub-population of individuals who had heard of antibiotics. With hindsight we would have been able to estimate the rest of our results (other than the question of antibiotic awareness) with more precision had we planned our sampled size to only include individuals who were aware of antibiotics, but while still collecting information on the key question of antibiotic awareness during an eligibility screening phase, for example. However, this does not affect the level of any bias in our results. A closely related limitation is that we sometimes ended up with impractically small sample sizes for some key questions that only small sub-populations within our sample could respond to. In terms of generalisability, the respondents remaining in our sub-population analyses are very unlikely to be representative of the complete rural communities that we sampled. Therefore, when generalising any of our sub-population results it is important to be clear that they can only be robustly applied to the relevant sub-populations within the sampled target population of adult (aged 18–49) female and male household heads, and the children of those females, within rural communities in Cumilla district. For example, our results on the frequency with which adult female household heads have previously taken antibiotics can only robustly be generalised to the population of adult female household heads living in rural communities who have also heard of antibiotics. Generalisations beyond this group would be questionable. The final obvious key limitation of the study is that we relied on self-reported responses, which are inevitably prone to respondent recall bias and social desirability bias. Obtaining more objective data in such surveys and contexts is very difficult, but future work could test possible methodological innovations [70].

## Conclusions

Our study provides rare, community-based data on antibiotic practices among humans and their domestic animals living in rural Bangladeshi communities. Given the lack of awareness about antibiotics and the human and animal practices currently identified that can lead to the development of antibiotic resistant microbes/infections, along with the risk that such practices will increase in the future, from a community perspective we argue that community-led, well informed and context-appropriate behaviour change education (BCE) is required as a first step. Our team has previously synthesised the key components necessary for delivering rural BCE on these issues in LMICs [71], and has explored appropriate and feasible approaches for delivering such a BCE in rural Bangladesh [72], and will further develop and evaluate a One Health focused BCE intervention to address these issues in rural Bangladesh. From a health system perspective there is an urgent need for much better regulation, monitoring and training of private providers of human healthcare, and far better provision of affordable, accessible and quality veterinary care for domestic animals. Finally, more community-based research is required to better understand the nature and scale of these issues across Bangladesh and other LMICs, to inform interventions and policy.

## Supplementary Information

**Additional file 1: Table S1.** Reported antibiotic awareness and use among household members. **Table S2.** Reported illnesses/main symptoms that have ever resulted in taking antibiotics. **Table S3.** Reported sources ever used to obtain antibiotics. **Table S4.** Reported behaviours relating to the WHO’s key recommended best practices for obtaining and using antibiotics for human illness when last obtaining and using antibiotics. **Table S5.** Reported current household ownership of domestic animals kept for small-scale husbandry. **Table S6.** Reported length of time households have owned poultry, cattle and/or goats/sheep that they currently own. **Table S7.** Reported risky animal husbandry practices and basic antibiotic use in households’ domestic animals. **Table S8.** Reported domestic animal illness and domestic animal treatment practices involving antibiotics. **Table S9.** Reported vaccination awareness, coverage and future preferences/desires.

## Data Availability

The datasets used and/or analysed during the current study are available from the corresponding author on reasonable request.

## Abbreviations

ABR: Antibiotic resistance
OIE: World Organisation for Animal Health
FAO: Food and Agriculture Organization of the United Nations
LMIC(s): Low- and middle-income country/countries
URTI(s): Upper respiratory tract infection(s)
HICs: High income countries
