# Supplementary material for: Antibiotic practices among household members and their domestic animals within rural communities in Cumilla district, Bangladesh: a cross-sectional survey
Source: BMC Public Health. 2021 Feb 25;21:406. doi: 10.1186/s12889-021-10457-w (PMC7908663; doi:10.1186/s12889-021-10457-w)
Supplement: Supplementary file 1 — Additional file 1: Table S1. Reported antibiotic awareness and use among household members. Table S2. Reported illnesses/main symptoms that have ever resulted in taking antibiotics. Table S3. Reported sources ever used to obtain antibiotics. Table S4. Reported behaviours relating to the WHO’s key recommended best practices for obtaining and using antibiotics for human illness when last obtaining and using antibiotics. Table S5. Reported current household ownership of domestic animals kept for small-scale husbandry. Table S6. Reported length of time households have owned poultry, cattle and/or goats/sheep that they currently own. Table S7. Reported risky animal husbandry practices and basic antibiotic use in households’ domestic animals. Table S8. Reported domestic animal illness and domestic animal treatment practices involving antibiotics. Table S9. Reported vaccination awareness, coverage and future preferences/desires. [file 12889_2021_10457_MOESM1_ESM.docx]

**Antibiotic practices among household members and their domestic animals within rural communities in Cumilla district, Bangladesh: a cross-sectional survey**

**Additional file 1**

**Authors**

Dr Joseph Paul Hicks (corresponding author)

Nuffield Centre for International Health and Development, University of Leeds, Leeds, LS2 9JT, UK

Email: j.p.hicks@leeds.ac.uk

Dr Sophia M Latham

Department of Livestock and One Health, Institute of Infection, Veterinary and Ecological Sciences, University of Liverpool, Leahurst Campus, Neston, Cheshire, CH64 7TE, UK

Professor Rumana Huque

ARK Foundation, Suite C-3 & C-4, House # 06, Road # 109, Gulshan-2, Dhaka-1212, Bangladesh

Department of Economics, University of Dhaka, Dhaka-1000, Bangladesh

Dr Mahua Das

Nuffield Centre for International Health and Development, University of Leeds, Leeds, LS2 9JT, UK

Jane Newell (Medical Student)

University of Liverpool Medical School, Cedar House, Ashton St, Liverpool, L69 3GE, UK

S. M. Abdullah

Department of Economics, University of Dhaka, Dhaka-1000, Bangladesh

ARK Foundation, Suite C-3 & C-4, House # 06, Road # 109, Gulshan-2, Dhaka-1212, Bangladesh

Zunayed Al Azdi

ARK Foundation, Suite C-3 & C-4, House # 06, Road # 109, Gulshan-2, Dhaka-1212, Bangladesh

Dr Ishrat Jahan

ARK Foundation, Suite C-3 & C-4, House # 06, Road # 109, Gulshan-2, Dhaka-1212, Bangladesh

Christian Rassi

Malaria Consortium, The Green House, 244-254 Cambridge Heath Road, London E2 9DA, UK

Dr Prudence Hamade

Malaria Consortium, The Green House, 244-254 Cambridge Heath Road, London E2 9DA, UK

Muhammad Shafique

Malaria Consortium, The Green House, 244-254 Cambridge Heath Road, London E2 9DA, UK

Mohammad Saiful Islam

Ex-office Dean. Faculty of Surgery and Professor of Paediatric Surgery, Bangabandhu Sheikh Mujib Medical University, Dhaka

Dr Rebecca King

Nuffield Centre for International Health and Development, University of Leeds, Leeds, LS2 9JT, UK

**Table S1. Reported antibiotic awareness and use among household members**

| **Question/response** | **Overall^a^** | **Women^a^** | **Men^a^** | **Children^a^** | **Men vs women difference^b^** | **Men vs children difference^b^** | **Women vs children difference^b^** |  |
| --- | --- | --- | --- | --- | --- | --- | --- | --- |
| Heard of ABs? |  |  |  |  |  |  |  |  |
| Yes | 623/1295 | 332/681 | 291/614 | - |  |  |  |  |
|  | 48% (40, 56) | 49% (40, 58) | 47% (39, 56) | - | -1PP (-7, 4) | - | - |  |
| No | 536/1295 | 292/681 | 244/614 | - |  |  |  |  |
|  | 41% (33, 51) | 43% (34, 52) | 40% (31, 50) | - | -3PP (-8, 2) | - | - |  |
| Don't know | 136/1295 | 57/681 | 79/614 | - |  |  |  |  |
|  | 11% (9, 13) | 8% (7, 10) | 13% (10, 16) | - | 4PP (2, 7) | - | - |  |
| Ever taken ABs? |  |  |  |  |  |  |  |  |
| Yes | 649/923 | 261/325 | 202/266 | 186/332 |  |  |  |  |
|  | 70% (64, 76) | 80% (76, 84) | 76% (68, 82) | 56% (45, 67) | -4PP (-9, 1) | 20PP (16, 30) | 24PP (16, 33) |  |
| No | 100/923 | 23/325 | 20/266 | 57/332 |  |  |  |  |
|  | 11% (7, 17) | 7% (4, 13) | 8% (4, 14) | 17% (11, 26) | 0PP (-4, 5) | -10PP (-15, -4) | -10PP (-15, -5) |  |
| Don't know | 174/923 | 41/325 | 44/266 | 89/332 |  |  |  |  |
|  | 19% (15, 23) | 13% (10, 16) | 17% (11, 24) | 27% (21, 33) | 4PP (-1, 9) | -10PP (-19, -4) | -14PP (-19, -9) |  |
| Last took ABs? |  |  |  |  |  |  |  |  |
| Never | 100/915 | 23/322 | 20/261 | 57/332 |  |  |  |  |
|  | 11% (7, 17) | 7% (4, 13) | 8% (4, 14) | 17% (11, 26) | 1PP (-4, 5) | -10PP (-15, -3) | -10PP (-15, -5) |  |
| <1 month | 196/915 | 76/322 | 53/261 | 67/332 |  |  |  |  |
|  | 21% (18, 25) | 24% (18, 30) | 20% (17, 25) | 20% (14, 28) | -3PP (-9, 3) | 0PP (-5, 6) | 3PP (-5, 12) |  |
| >1m & <6m | 255/915 | 109/322 | 73/261 | 73/332 |  |  |  |  |
|  | 28% (21, 35) | 34% (25, 44) | 28% (23, 33) | 22% (14, 33) | -6PP (-13, 2) | 6PP (5, 13) | 12PP (5, 19) |  |
| >6m | 190/915 | 73/322 | 71/261 | 46/332 |  |  |  |  |
|  | 21% (18, 24) | 23% (16, 30) | 27% (22, 33) | 14% (10, 19) | 5PP (-4, 13) | 13PP (1, 20) | 9PP (1, 17) |  |
| Don't know | 174/915 | 41/322 | 44/261 | 89/332 |  |  |  |  |
|  | 19% (15, 23) | 13% (10, 16) | 17% (11, 25) | 27% (21, 33) | 4PP (-1, 9) | -10PP (-19, -3) | -14PP (-19, -9) |  |
| AB = antibiotic.  ^a^Values are n/total and % (95% CI), where n = number of individuals reporting the relevant response and total = number of individuals eligible to respond to the question given prior responses. 95% CIs are calculated via a "logit" method: using a logistic regression model the 95% CI are computed on the log-odds scale, based on the Wald statistic, and are then transformed to the probability scale. 95% CIs are also adjusted for the clustered and stratified sampling. The “overall” results are based on the combined responses from all female and male respondents, and (if applicable) the responses of female respondents on behalf of any children (under 15) they have collectively.  ^b^Differences are calculated as percentage points (PP) using binomial regression models (i.e. generalised linear models with identity links and binomial errors): each response is treated as a binary outcome and a single independent (dummy) variable codes for the relevant comparison, with models accounting for the clustered and stratified sampling. | | | | | | | | |

Table S2. Reported illnesses/main symptoms that have ever resulted in taking antibiotics

| **Illness/main symptoms** | **Overall^a^** | **Women^a^** | **Men^a^** | **Children^a^** | **Men vs women difference^b^** | **Men vs children difference^b^** | **Women vs children difference^b^** |
| --- | --- | --- | --- | --- | --- | --- | --- |
| Fever | 434/638 | 171/254 | 124/199 | 139/185 |  |  |  |
|  | 68% (60, 75) | 67% (58, 76) | 62% (53, 71) | 75% (61, 85) | -5PP (-12, 2) | -13PP (-25, -1) | -8PP (-19, 3) |
| Cough/cold | 329/638 | 117/254 | 96/199 | 116/185 |  |  |  |
|  | 52% (40, 63) | 46% (34, 58) | 48% (35, 62) | 63% (48, 75) | 2PP (-10, 14) | -14PP (-24, -5) | -17PP (-26, -8) |
| Headache/body pain | 222/638 | 111/254 | 85/199 | 26/185 |  |  |  |
|  | 35% (27, 44) | 44% (32, 56) | 43% (30, 57) | 14% (9, 22) | -1PP (-7, 5) | 29PP (14, 43) | 30PP (18, 42) |
| Sore throat | 153/638 | 74/254 | 39/199 | 40/185 |  |  |  |
|  | 24% (19, 30) | 29% (21, 38) | 20% (14, 27) | 22% (16, 29) | -10PP (-17, -2) | -2PP (-10, 6) | 8PP (1, 14) |
| Skin/wound infection | 117/638 | 59/254 | 31/199 | 27/185 |  |  |  |
|  | 18% (16, 20) | 23% (19, 28) | 16% (11, 22) | 15% (9, 22) | -8PP (-16, 1) | 1PP (-8, 10) | 9PP (1, 16) |
| Fatigue | 93/638 | 52/254 | 31/199 | 10/185 |  |  |  |
|  | 15% (10, 21) | 20% (13, 31) | 16% (10, 24) | 5% (3, 10) | -5PP (-11, 2) | 10PP (3, 17) | 15PP (7, 23) |
| Diarrhoea | 75/638 | 18/254 | 15/199 | 42/185 |  |  |  |
|  | 12% (9, 15) | 7% (5, 11) | 8% (5, 11) | 23% (17, 30) | 0PP (-4, 5) | -15PP (-21, -10) | -16PP (-22, -9) |
| Vomiting | 60/638 | 24/254 | 13/199 | 23/185 |  |  |  |
|  | 9% (6, 14) | 9% (6, 14) | 7% (3, 15) | 12% (7, 22) | -3PP (-8, 2) | -6PP (-13, 1) | -3PP (-8, 2) |
| Cough w/ fast breathing | 45/638 | 2/254 | 15/199 | 28/185 |  |  |  |
|  | 7% (6, 9) | 1% (0, 4) | 8% (4, 14) | 15% (11, 21) | 7PP (2, 11) | -8PP (-15, -1) | -14PP (-18, -11) |
| Weight loss | 25/638 | 12/254 | 2/199 | 11/185 |  |  |  |
|  | 4% (3, 5) | 5% (3, 8) | 1% (0, 5) | 6% (3, 11) | -4PP (-6, -2) | -5PP (-9, -1) | -1PP (-6, 4) |
| Measles | 11/638 | 2/254 | 4/199 | 5/185 |  |  |  |
|  | 2% (1, 3) | 1% (0, 4) | 2% (1, 4) | 3% (1, 6) | 1PP (-1, 3) | -1PP (-3, 1) | -2PP (-4, 0) |
| Other | 72/638 | 42/254 | 22/199 | 8/185 |  |  |  |
|  | 11% (7, 18) | 17% (9, 29) | 11% (6, 19) | 4% (3, 7) | -5PP (-12, 1) | 7PP (2, 12) | 12PP (3, 21) |
| Don't know | 7/638 | 2/254 | 3/199 | 2/185 |  |  |  |
|  | 1% (0, 3) | 1% (0, 3) | 2% (0, 8) | 1% (0, 5) | 1PP (-2, 3) | 0PP (-1, 2) | 0PP (-2, 2) |
| ^a^Values are n/total and % (95% CI), where n = number of individuals reporting the relevant response and total = number of individuals eligible to respond to the question given prior responses, with any missing responses excluded. 95% CIs are calculated via a "logit" method: using a logistic regression model the 95% CI are computed on the log-odds scale, based on the Wald statistic, and are then transformed to the probability scale. 95% CIs are also adjusted for the clustered and stratified sampling. The “overall” results are based on the combined responses from all female and male respondents, and the responses of female respondents on behalf of any children (under 15) they have collectively.  ^b^Differences are calculated as percentage points (PP) using binomial regression models (i.e. generalised linear models with identity links and binomial errors): each reported illness/main symptom is treated as a binary outcome and a single independent (dummy) variable codes for the relevant comparison, with models accounting for the clustered and stratified sampling. | | | | | | | |

Table S3. Reported sources ever used to obtain antibiotics

| **Antibiotic source** | **Overall^a^** | **Women^a^** | **Men^a^** | **Children^a^** | **Men vs women difference^b^** | **Men vs children difference^b^** | **Women vs children difference^b^** |  |
| --- | --- | --- | --- | --- | --- | --- | --- | --- |
| Pharmacy | 502/637 | 211/257 | 152/194 | 139/186 |  |  |  |  |
|  | 79% (73, 84) | 82% (76, 87) | 78% (66, 87) | 75% (64, 83) | -4PP (-13, 5) | 4PP (-10, 17) | 7PP (-1, 15) |  |
| PMP | 224/637 | 87/257 | 69/194 | 68/186 |  |  |  |  |
|  | 35% (27, 44) | 34% (23, 47) | 36% (25, 47) | 37% (28, 46) | 2PP (-8, 12) | -1PP (-12, 10) | -3PP (-14, 9) |  |
| Paramedic/village doctor | 125/637 | 59/257 | 26/194 | 40/186 |  |  |  |  |
|  | 20% (14, 27) | 23% (16, 32) | 13% (8, 23) | 22% (15, 30) | -10PP (-15, -4) | -8PP (-16, 0) | 1PP (-3, 6) |  |
| UHC | 90/637 | 31/257 | 28/194 | 31/186 |  |  |  |  |
|  | 14% (9, 21) | 12% (8, 18) | 14% (8, 26) | 17% (9, 29) | 2PP (-3, 8) | -2PP (-11, 7) | -5PP (-13, 4) |  |
| CC | 35/637 | 19/257 | 4/194 | 12/186 |  |  |  |  |
|  | 5% (2, 12) | 7% (3, 17) | 2% (1, 8) | 6% (2, 18) | -5PP (-12, 1) | -4PP (-11, 2) | 1PP (-2, 4) |  |
| Traditional healer | 24/637 | 10/257 | 2/194 | 12/186 |  |  |  |  |
|  | 4% (3, 5) | 4% (2, 7) | 1% (0, 4) | 6% (5, 8) | -3PP (-5, 0) | -5PP (-7, -3) | -3PP (-5, 0) |  |
| Family/neighbours | 17/637 | 3/257 | 3/194 | 11/186 |  |  |  |  |
|  | 3% (2, 4) | 1% (1, 2) | 2% (0, 5) | 6% (4, 9) | 0PP (-1, 2) | -4PP (-6, -2) | -5PP (-7, -3) |  |
| HA/UHFWC/DH/NGO | 37/636 | 21/257 | 9/193 | 7/186 |  |  |  |  |
|  | 6% (3, 10) | 8% (4, 16) | 5% (2, 9) | 4% (1, 10) | -4PP (-7, 0) | 1PP (-3, 5) | 4PP (-1, 10) |  |
| Don't know | 1/637 | 0/257 | 0/194 | 1/186 |  |  |  |  |
|  | 0% (0, 1) | 0%^c^ | 0%^c^ | 1% (0, 4) | –^c^ | –^c^ | –^c^ |  |
| Pharmacy: private pharmacy with qualified pharmacist/informal and unqualified drug seller. PMP = private medical practitioner: a medically trained doctor operating privately. Paramedic/village doctor: an individual having medical training but less than a medical doctor/an untrained “quack”, operating privately. UHC = upazila health complex: a sub-district level, public, hospital, staffed by medical doctors and other cadres. CC = community clinic: a community-level, public, primary-care clinic, staffed by a paramedic. Traditional healer: a “quack” primarily practicing “traditional healing” methods. HA = health assistant: a public community health worker. UHFWC = union health and family welfare centre: a union-level (between sub-district and community levels), public primary care facility focusing on maternal and child health, staffed by paramedics and health workers. DH = district (or general) hospital: a district-level public hospital providing secondary care, staffed by medical doctors and other cadres. NGO = non-governmental donor funded health facility: a local or higher level facility providing primary and/or secondary care, staffed by private medical doctors and other cadres.  ^a^Values are n/total and % (95% CI), where n = number of individuals reporting the relevant response and total = number of individuals eligible to respond to the question given prior responses, with any missing responses excluded. 95% CIs are calculated via a "logit" method: using a logistic regression model the 95% CI are computed on the log-odds scale, based on the Wald statistic, and are then transformed to the probability scale. 95% CIs are also adjusted for the clustered and stratified sampling. The “overall” results are based on the combined responses from all female and male respondents, and the responses of female respondents on behalf of any children (under 15) they have collectively.  ^b^Differences are calculated as percentage points (PP) using binomial regression models (i.e. generalised linear models with identity links and binomial errors): each reported AB source is treated as a binary outcome and a single independent (dummy) variable codes for the relevant comparison, with models accounting for the clustered and stratified sampling.  ^c^Confidence intervals or comparison estimates not computable due to outcome proportion(s) being exactly 0%. | | | | | | | | |

Table S4. Reported behaviours relating to the WHO’s key recommended best practices for obtaining and using antibiotics for human illness when last obtaining and using antibiotics

| **Recommended/reported practice** | **Overall^a^** | **Women^a^** | **Men^a^** | **Children^a^** | **Men vs women difference^b^** | **Men vs children difference^b^** | **Women vs children difference^b^** |  |
| --- | --- | --- | --- | --- | --- | --- | --- | --- |
| Required a prescription to buy antibiotics? |  |  |  |  |  |  |  |  |
| Yes | 441/640 | 162/259 | 142/195 | 137/186 |  |  |  |  |
|  | 69% (61, 76) | 63% (52, 72) | 73% (63, 81) | 74% (60, 84) | 10PP (0, 20) | -1PP (-23, 13) | -11PP (-23, 1) |  |
| No | 192/640 | 95/259 | 50/195 | 47/186 |  |  |  |  |
|  | 30% (24, 37) | 37% (28, 46) | 26% (18, 35) | 25% (15, 39) | -11PP (-22, 0) | 0PP (0, 14) | 11PP (0, 23) |  |
| Don't know | 7/640 | 2/259 | 3/195 | 2/186 |  |  |  |  |
|  | 1% (0, 3) | 1% (0, 6) | 2% (0, 5) | 1% (0, 5) | 1PP (-1, 2) | 0PP (-2, 3) | 0PP (-2, 2) |  |
| Given the full course of antibiotics? |  |  |  |  |  |  |  |  |
| Yes | 549/631 | 221/259 | 160/186 | 168/186 |  |  |  |  |
|  | 87% (79, 92) | 85% (69, 94) | 86% (78, 92) | 90% (81, 95) | 1PP (-12, 13) | -4PP (-14, 4) | -5PP (-14, 4) |  |
| No | 41/631 | 20/259 | 16/186 | 5/186 |  |  |  |  |
|  | 6% (4, 10) | 8% (3, 17) | 9% (5, 14) | 3% (1, 8) | 1PP (-8, 10) | 6PP (0, 12) | 5PP (0, 10) |  |
| Don't know | 41/631 | 18/259 | 10/186 | 13/186 |  |  |  |  |
|  | 6% (3, 15) | 7% (2, 24) | 5% (2, 13) | 7% (3, 14) | -2PP (-9, 6) | -2PP (-8, 2) | 0PP (-8, 8) |  |
| Finished the full course of antibiotics? |  |  |  |  |  |  |  |  |
| Yes | 469/541 | 185/214 | 141/159 | 143/168 |  |  |  |  |
|  | 87% (81, 91) | 86% (81, 90) | 89% (72, 96) | 85% (79, 89) | 2PP (-8, 12) | 4PP (-2, 14) | 1PP (-2, 5) |  |
| No | 63/541 | 26/214 | 18/159 | 19/168 |  |  |  |  |
|  | 12% (7, 18) | 12% (8, 17) | 11% (4, 28) | 11% (7, 18) | -1PP (-11, 9) | 0PP (-3, 9) | 1PP (-3, 4) |  |
| Don't know | 9/541 | 3/214 | 0/159 | 6/168 |  |  |  |  |
|  | 2% (1, 5) | 1% (0, 6) | 0%^c^ | 4% (1, 9) | –^c^ | –^c^ | -2PP (-5, 0) |  |
| Took the recommended dose of antibiotics? |  |  |  |  |  |  |  |  |
| Recommended | 516/638 | 202/256 | 159/196 | 155/186 |  |  |  |  |
|  | 81% (74, 86) | 79% (70, 86) | 81% (68, 90) | 83% (75, 89) | 2PP (-9, 13) | -2PP (-11, 6) | -4PP (-11, 2) |  |
| Fewer | 107/638 | 44/256 | 37/196 | 26/186 |  |  |  |  |
|  | 17% (12, 23) | 17% (11, 25) | 19% (10, 32) | 14% (10, 20) | 2PP (-9, 12) | 5PP (-2, 13) | 3PP (-2, 9) |  |
| More | 8/638 | 6/256 | 0/196 | 2/186 |  |  |  |  |
|  | 1% (0, 3) | 2% (1, 6) | 0%^c^ | 1% (0, 5) | –^c^ | –^c^ | –^d^ |  |
| Don't know/not advised | 7/638 | 4/256 | 0/196 | 3/186 |  |  |  |  |
|  | 1% (0, 3) | 2% (1, 5) | 0%^c^ | 2% (0, 8) | –^c^ | –^c^ | –^d^ |  |
| Ever taken leftover antibiotics? |  |  |  |  |  |  |  |  |
| Yes | 38/560 | 27/256 | 1/197 | 10/107 |  |  |  |  |
|  | 7% (4, 11) | 11% (6, 18) | 1% (0, 5) | 9% (4, 20) | -10PP (-15, -5) | -9PP (-6, -3) | 1PP (-6, 9) |  |
| No | 516/560 | 225/256 | 195/197 | 96/107 |  |  |  |  |
|  | 92% (88, 95) | 88% (82, 92) | 99% (95, 100) | 90% (79, 95) | 11PP (6, 17) | 9PP (-10, 16) | -2PP (-10, 6) |  |
| Don't know | 6/560 | 4/256 | 1/197 | 1/107 |  |  |  |  |
|  | 1% (0, 3) | 2% (0, 5) | 1% (0, 5) | 1% (0, 8) | -1PP (-3, 1) | 0PP (-2, 2) | 1PP (-2, 3) |  |
| WHO recommended best practices when obtaining and using antibiotics for human illness: 1) Only use antibiotics when prescribed by a certified health professional. 2) Never demand antibiotics if your health worker says you don’t need them. 3) Always follow your health worker’s advice when using antibiotics. 4) Never share or use leftover antibiotics. Taken from: World Health Organization, Food and Agriculture Organization of the United Nations & World Organisation for Animal Health. 2016. World antibiotic awareness week 14-20 November 2016: 2016 campaign toolkit. Geneva: World Health Organization. <https://www.who.int/campaigns/world-antibiotic-awareness-week/Toolkit2016.pdf>  Required a prescription to buy antibiotics? = did you require a prescription to buy antibiotics the last time you obtained them?  Given the full course of antibiotics? = were you given the full course when you last obtained antibiotics?  Finished the full course of antibiotics? = did you finish the full course when you last obtained antibiotics (only asked if the respondent/child was given a full course)?  Took the recommended dose of antibiotics? = did you/your child take the dose (e.g. daily number of tablets) as recommended by the health practitioner that you obtained the antibiotics from?  Ever taken leftover antibiotics? = have you ever taken antibiotics leftover from treating a previous illness (either your own or someone else's illness)?  ^a^Values are n/total and % (95% CI), where n = number of individuals reporting the relevant response and total = number of individuals eligible to respond to the question given prior responses, with any missing responses excluded. 95% CIs are calculated via a "logit" method: using a logistic regression model the 95% CI are computed on the log-odds scale, based on the Wald statistic, and are then transformed to the probability scale. 95% CIs are also adjusted for the clustered and stratified sampling. The “overall” results are based on the combined responses from all female and male respondents, and the responses of female respondents on behalf of any children (under 15) they have collectively.  ^b^Differences are calculated as percentage points (PP) using binomial regression models (i.e. generalised linear models with identity links and binomial errors): each reported behaviour is treated as a binary outcome and a single independent (dummy) variable codes for the relevant comparison, with models accounting for the clustered and stratified sampling.  ^c^Confidence intervals and/or difference estimates not computable due to outcome proportion(s) being exactly 0%.  ^d^Difference estimates not computable due to model non-convergence. | | | | | | | | |

Table S5. Reported current household ownership of domestic animals kept for small-scale husbandry

| **Animal group** | **% (95% CI)^a^** | **n/total^b^** | **Mean no. of animals owned (95% CI)^c^** |
| --- | --- | --- | --- |
| Any poultry | 73% (68, 78) | 499/682 | 10 (9, 11) |
| Chickens | 69% (64, 74) | 471/682 | 6 (6, 7) |
| Ducks | 47% (40, 55) | 321/682 | 5 (5, 6) |
| Geese | 6% (4, 8) | 39/682 | 3 (3, 4) |
| Any bovids | 37% (31, 43) | 228/620 | 3 (2, 3) |
| Cattle | 32% (26, 40) | 200/620 | 2 (2, 3) |
| Goats/sheep | 9% (6, 13) | 54/620 | 3 (2, 4) |
| ^a^95% CIs are calculated via a "logit" method: using a logistic regression model the 95% CI are computed on the log-odds scale, based on the Wald statistic, and are then transformed to the probability scale. 95% CIs are also adjusted for the clustered and stratified sampling.  ^b^Total = all female respondents for poultry group, and all male respondents for bovid group.  ^c^Calculated means exclude all households with no ownership of the relevant animal group. | | | |

**Table S6. Reported length of time households have owned poultry, cattle and/or goats/sheep that they currently own**

|  | **Poultry** | | **Cattle** | | **Goats/sheep** | |
| --- | --- | --- | --- | --- | --- | --- |
| **Length of ownership** | **% (95% CI)^a^** | **n/total^b^** | **% (95% CI)^a^** | **n/total^b^** | **% (95% CI)^a^** | **n/total^b^** |
| Less than 1 year | 37% (31, 44) | 186/498 | 27% (18, 39) | 50/187 | 46% (27, 66) | 23/50 |
| Between 1 and 5 years | 27% (23, 30) | 132/498 | 37% (27, 49) | 70/187 | 42% (24, 62) | 21/50 |
| More than 5 years | 32% (26, 38) | 157/498 | 32% (24, 41) | 60/187 | 12% (4, 31) | 6/50 |
| Don’t know | 5% (2, 8) | 23/498 | 4% (1, 14) | 7/187 | 0% | 0/50 |
| ^a^95% CIs are calculated via a "logit" method: using a logistic regression model the 95% CI are computed on the log-odds scale, based on the Wald statistic, and are then transformed to the probability scale. 95% CIs are also adjusted for the clustered and stratified sampling.  ^b^Total = number of individuals owning relevant animal type, among female respondents for poultry and male respondents for cattle and goats/sheep. | | | | | | |

Table S7. Reported risky animal husbandry practices and basic antibiotic use in households’ domestic animals

|  | | **Poultry** | | | **Cattle** | |  | | |
| --- | --- | --- | --- | --- | --- | --- | --- | --- | --- |
| **Question/response** | **% (95% CI)^a^** | | **n/total^b^** | **% (95% CI)^a^** | | **n/total^b^** | | **Poultry vs cattle difference^c^** |  |
| Where are your animals kept at night? |  | |  |  | |  | |  |  |
| Inside the house | 76% (68, 83) | | 363/475 | 35% (28, 43) | | 66/188 | | -41PP (-49, -34) |  |
| Outside the house | 23% (16, 31) | | 107/475 | 61% (51, 69) | | 114/188 | | 38PP (28, 48) |  |
| Other | 1% (0, 4) | | 5/475 | 4% (1, 13) | | 8/188 | | 3PP (-1, 8) |  |
| Do your animals ever share your cooking/bathing water? |  | |  |  | |  | |  |  |
| Yes | 42% (35, 49) | | 204/486 | 49% (35, 64) | | 96/194 | | 8PP (-7, 22) |  |
| No | 58% (51, 65) | | 282/486 | 51% (36, 65) | | 98/194 | | -8PP (-22, 7) |  |
| Have you ever given ABs to your animals when ill? |  | |  |  | |  | |  |  |
| Yes | 24% (11, 45) | | 21/86 | 36% (13, 67) | | 10/28 | | -11PP (-24, 2) |  |
| No | 37% (24, 53) | | 32/86 | 11% (2, 43) | | 3/28 | | 26PP (7, 46) |  |
| Don't know | 38% (27, 52) | | 32/86 | 54% (17, 87) | | 15/28 | | -15PP (-44, 14) |  |
| Have you ever given ABs to your animals when healthy? |  | |  |  | |  | |  |  |
| Yes | 11% (8, 15) | | 29/253 | 2% (0, 9) | | 2/115 | | 10PP (6, 13) |  |
| No | 85% (81, 89) | | 216/253 | 96% (90, 98) | | 110/115 | | -10PP (-14, -6) |  |
| Don't know | 3% (1, 9) | | 8/253 | 3% (1, 6) | | 3/115 | | 1PP (-3, 4) |  |
| Have you ever bought animal feed with ABs in? |  | |  |  | |  | |  |  |
| Sometimes/usually/always | 21% (7, 50) | | 7/33 | 20% (9, 37) | | 13/66 | | 2PP (-20, 23) |  |
| Never | 48% (21, 77) | | 16/33 | 48% (40, 57) | | 32/66 | | 0PP (-23, 23) |  |
| Don't know | 30% (11, 61) | | 10/33 | 32% (17, 51) | | 21/66 | | -2PP (-17, 14) |  |
| ABs = antibiotics.  ^a^95% CIs are calculated via a "logit" method: using a logistic regression model the 95% CI are computed on the log-odds scale, based on the Wald statistic, and are then transformed to the probability scale. 95% CIs are also adjusted for the clustered and stratified sampling.  ^b^Total = number of individuals eligible to respond given prior responses, with any missing responses excluded.  ^c^Differences are calculated as percentage points (PP) using binomial regression models (i.e. generalised linear models with identity links and binomial errors): each response is treated as a binary outcome and a single independent (dummy) variable codes for the relevant comparison, with models accounting for the clustered and stratified sampling. | | | | | | | | | |

**Table S8. Reported domestic animal illness and domestic animal treatment practices involving antibiotics**

|  | **Poultry** | | **Cattle** | |  |
| --- | --- | --- | --- | --- | --- |
| **Question/response** | **% (95% CI)^a^** | **n/total^b^** | **% (95% CI)^a^** | **n/total^b^** | **Poultry vs cattle difference^c^** |
| Ever had any poultry/cattle become ill/die? |  |  |  |  |  |
| Yes | 65% (53, 75) | 303/466 | 27% (17, 39) | 47/176 | 38PP (30, 47) |
| No | 35% (25, 47) | 163/466 | 73% (61, 83) | 129/176 | -38PP (-47, -30) |
| Last poultry/cattle illness/death? |  |  |  |  |  |
| ≤6 months ago | 77% (69, 84) | 228/295 | 57% (39, 73) | 26/46 | 21PP (7, 34) |
| >6 months ago | 23% (16, 31) | 67/295 | 41% (24, 61) | 19/46 | -19PP (-33, -4) |
| Don't know | 0%^d^ | 0/295 | 2% (0, 20) | 1/46 | –^e^ |
| What actions have you ever taken for any of your ill poultry/cattle? |  |  |  |  |  |
| Sought treatment | 47% (37, 58) | 141/299 | 94% (77, 98) | 44/47 | -46PP (-59, -34) |
| Killed/sold them | 33% (24, 44) | 100/299 | 6% (2, 23) | 3/47 | 27PP (15, 39) |
| Left them alone | 15% (10, 21) | 44/299 | 0%^d^ | 0/47 | –^e^ |
| Don't know | 5% (2, 9) | 14/299 | 0%^d^ | 0/47 | –^e^ |
| What treatments have any of your ill poultry/cattle ever had? |  |  |  |  |  |
| Traditional “home” or “folk” remedies/homeopathy | 16% (10, 24) | 22/141 | 7% (1, 30) | 3/44 | 9PP (-1, 19) |
| Drugs^d^ | 75% (60, 86) | 106/141 | 98% (79, 100) | 43/44 | -23PP (-33, -12) |
| Vitamins | 1% (0, 6) | 1/141 | 23% (8, 49) | 10/44 | -22PP (-38, -6) |
| Other | 62% (38, 81) | 87/141 | 100%^d^ | 44/44 | –^e^ |
| Don't know | 4% (2, 10) | 6/141 | 2% (0, 24) | 1/44 | 2PP (-4, 8) |
| Who has ever given these treatments to your ill poultry/cattle? |  |  |  |  |  |
| Household member/neighbour | 33% (18, 53) | 47/141 | 23% (10, 43) | 10/44 | 11PP (-3, 25) |
| Village elder/traditional healer | 9% (4, 17) | 12/141 | 5% (1, 23) | 2/44 | 4PP (-5, 13) |
| Village doctor/paramedic/PMP | 17% (7, 35) | 24/141 | 27% (19, 38) | 12/44 | -10PP (-22, 1) |
| Drug seller/pharmacist | 48% (35, 60) | 67/141 | 14% (5, 31) | 6/44 | 34PP (24, 43) |
| Government vet | 1% (0, 7) | 1/141 | 25% (14, 41) | 11/44 | -24PP (-35, -13) |
| Private vet | 4% (1, 18) | 5/141 | 32% (12, 62) | 14/44 | -28PP (-48, -9) |
| Other | 62% (37, 82) | 88/141 | 100%^d^ | 44/44 | –^e^ |
| Don't know | 1% (0, 7) | 1/141 | 0%^d^ | 0/44 | –^e^ |
| Have your ill poultry/cattle ever been given ABs? |  |  |  |  |  |
| Yes | 24% (11, 45) | 21/86 | 36% (13, 67) | 10/28 | -11PP (-24, 2) |
| No | 37% (24, 53) | 32/86 | 11% (2, 43) | 3/28 | 26PP (7, 46) |
| Don't know | 38% (27, 52) | 32/86 | 54% (17, 87) | 15/28 | -15PP (-44, 14) |
| Where have these antibiotics ever been obtained from? |  |  |  |  |  |
| Household member/neighbour | 10% (0, 71) | 2/21 | 0%^d^ | 0/10 | –^e^ |
| Village elder/traditional healer | 0%^d^ | 0/21 | 20% (5, 53) | 2/10 | –^e^ |
| Pharmacist/drug seller | 95% (17, 100) | 20/21 | 0%^d^ | 0/10 | –^e^ |
| Village doctor/paramedic/PMP | 5% (0, 76) | 1/21 | 60% (7, 97) | 6/10 | -55PP (-92, -18) |
| Government vet | 0%^d^ | 0/21 | 30% (2, 92) | 3/10 | –^e^ |
| Private vet | 5% (0, 80) | 1/21 | 0%^d^ | 0/10 | –^e^ |
| Other | 29% (4, 79) | 6/21 | 100%^d^ | 10/10 | –^e^ |
| Don't know | 0%^d^ | 0/21 | 0%^d^ | 0/10 | –^e^ |
| Have your poultry/cattle ever been given ABs when healthy? |  |  |  |  |  |
| Yes | 11% (8, 15) | 29/253 | 2% (0, 9) | 2/115 | 10PP (6, 13) |
| No | 85% (81, 89) | 216/253 | 96% (90, 98) | 110/115 | -10PP (-14, -6) |
| Don't know | 3% (1, 9) | 8/253 | 3% (1, 6) | 3/115 | 1PP (-3, 4) |
| Why have your poultry/cattle ever been given ABs when healthy? |  |  |  |  |  |
| Increase/speed-up produce (meat/eggs/milk) | 45% (12, 82) | 13/29 | 50%^e^ | 1/2 | –^f^ |
| Prevent disease | 97% (48, 100) | 28/29 | 100%^e^ | 2/2 | –^f^ |
| Other | 0%^d^ | 0/29 | 100%^e^ | 2/2 | –^f^ |
| Don't know | 0%^d^ | 0/29 | 0%^e^ | 0/2 | –^f^ |
| Ever bought feed for your poultry/cattle? |  |  |  |  |  |
| Sometimes/usually/always | 14% (9, 23) | 35/245 | 63% (41, 81) | 67/106 | -49PP (-65, -33) |
| Never | 86% (77, 91) | 210/245 | 36% (19, 58) | 38/106 | 50PP (35, 65) |
| Don't know | 0%^d^ | 0/245 | 1% (0, 8) | 1/106 | –^e^ |
| Ever bought feed containing ABs for your poultry/cattle? |  |  |  |  |  |
| Sometimes/usually/always | 21% (7, 50) | 7/33 | 20% (9, 37) | 13/66 | 2PP (-20, 23) |
| Never | 48% (21, 77) | 16/33 | 48% (40, 57) | 32/66 | 0PP (-23, 23) |
| Don't know | 30% (11, 61) | 10/33 | 32% (17, 51) | 21/66 | -2PP (-17, 14) |
| Note: all questions were asked of female and male respondents who previously indicated that their household owns poultry or cattle respectively, with female respondents asked about household poultry and only responding in relation to household poultry and male respondents asked about household cattle and responding in relation to household cattle only.  PMP = private medical practitioner: a medically trained doctor operating privately.  ABs = antibiotics.  ^a^95% CIs are calculated via a "logit" method: using a logistic regression model the 95% CI are computed on the log-odds scale, based on the Wald statistic, and are then transformed to the probability scale. 95% CIs are also adjusted for the clustered and stratified sampling.  ^b^Total = number of individuals eligible to respond given prior responses.  ^c^Differences are calculated as percentage points (PP) using binomial regression models (i.e. generalised linear models with identity links and binomial errors): each response is treated as a binary outcome and a single independent (dummy) variable codes for the relevant comparison, with models accounting for the clustered and stratified sampling.  ^d^Drugs = includes food with added medicine/drugs, antihelmintics/antiparasite drugs, pill/liquid western medicines, and/or injected western medicines.  ^e^Confidence intervals and/or difference estimates not computable due to outcome proportion(s) being exactly 0%/100%.  ^f^Difference estimates not computable due to model non-convergence. | | | | | |

**Table S9. Reported vaccination awareness, coverage and future preferences/desires**

|  | **Female respondents / in relation to household poultry** | | **Male respondents / in relation to household cattle** | |  |
| --- | --- | --- | --- | --- | --- |
| **Question/response** | **% (95% CI)** | **n/total** | **% (95% CI)** | **n/total** | **Female/poultry vs male/cattle difference** |
| Have you heard of vaccination? |  | |  | |  |
| Yes | 35% (28, 44) | 176/498 | 76% (61, 86) | 136/179 | -41PP (-48, -33) |
| No | 52% (45, 60) | 261/498 | 21% (12, 34) | 37/179 | 32PP (23, 40) |
| Don't know | 12% (9, 17) | 61/498 | 3% (2, 7) | 6/179 | –^d^ |
| Are any of your poultry/cattle vaccinated? |  | |  | |  |
| All | 2% (0, 7) | 3/163 | 6% (3, 14) | 8/124 | -5PP (-10, 1) |
| Few | 9% (5, 17) | 15/163 | 17% (11, 26) | 21/124 | -8PP (-15, 0) |
| None | 82% (75, 88) | 134/163 | 71% (60, 80) | 88/124 | 11PP (3, 19) |
| Don't know | 7% (4, 11) | 11/163 | 6% (3, 11) | 7/124 | 1PP (-3, 5) |
| If possible would you like more of your poultry/cattle vaccinated? |  | |  | |  |
| Yes | 77% (66, 86) | 136/176 | 83% (59, 94) | 34/41 | -6PP (-19, 8) |
| No | 20% (13, 31) | 36/176 | 15% (4, 39) | 6/41 | 6PP (-7, 19) |
| Don't know | 2% (0, 11) | 4/176 | 2% (0, 19) | 1/41 | 0PP (-2, 2) |
| What is preventing you from having more of your poultry/cattle vaccinated? |  | |  | |  |
| Cost | 80% (69, 87) | 51/64 | 80% (58, 92) | 20/25 | 0PP (-16, 16) |
| Lack of access (professionals/facilities) | 66% (51, 78) | 42/64 | 40% (20, 65) | 10/25 | 26PP (3, 48) |
| Don't know | 22% (15, 31) | 14/64 | 68% (40, 87) | 17/25 | -46PP (-68, -24) |
| All questions were asked of female and male respondents who previously indicated that their household owns poultry or cattle respectively, with female respondents asked about household poultry only and male respondents asked about household cattle only.  ^a^95% CIs are calculated via a "logit" method: using a logistic regression model the 95% CI are computed on the log-odds scale, based on the Wald statistic, and are then transformed to the probability scale. 95% CIs are also adjusted for the clustered and stratified sampling.  ^b^Total = number of individuals eligible to respond given prior responses.  ^c^Differences are calculated as percentage points (PP) using binomial regression models (i.e. generalised linear models with identity links and binomial errors): each response is treated as a binary outcome and a single independent (dummy) variable codes for the relevant comparison, with models accounting for the clustered and stratified sampling.  ^d^Difference estimates not computable due to model non-convergence. | | | | | |
